# Evaluating interdisciplinary breastfeeding and lactation knowledge, attitudes and skills: An evaluation of a professional graduate programme for healthcare professionals

**DOI:** 10.1371/journal.pone.0310500

**Published:** 2025-01-31

**Authors:** Denise McGuinness, Kate Frazer, Karl F. Conyard, Paula Cornally, Lauren Cooper, Niamh Vickers

**Affiliations:** 1 School of Nursing, Midwifery & Health Systems University College, Dublin, Ireland; 2 School of Public Health, Physiotherapy and Sports Science, Dublin, Ireland; 3 The Royal College of Surgeons in Ireland, University of Medicine and Health Science, School of Medicine, Dublin, Ireland; University of Oulu: Oulun Yliopisto, FINLAND

## Abstract

Breastfeeding theoretical and skills training is important for health care professionals engaging with the mother infant dyad to increase breastfeeding exclusivity and duration. The aim of this study was to evaluate the knowledge, attitudes and practices (KAPs) of health care professionals following completion of a university professional graduate programme in breastfeeding and lactation. A pre and post—educational study design was used. All students enrolled in a six month programme were invited to complete an online anonymous survey at two time points: January 2023 and July 2023. Ethical approval (LS-C-23-17) was obtained in January 2023. Descriptive statistics were utilised to report percentages and means, and independent T tests were used to report mean differences between variables on knowledge, attitude and practices. All students completed the module. The pre survey participant response rate was n = 55 (92.82%) and the post survey participant response rate n = 33 (60%). Comparison of the pre and post questionnaire report nine statistically significant results following completion of the university breastfeeding and lactation programme. Knowledge scores increased specifically with higher mean knowledge scores for reporting “I am confident with my knowledge about breastfeeding” and statistically significant mean difference of 0.29 following completion of the module (95% CI, 0.13 to 0.45) (t (64) = 3.59, p = 0.001). The programme evaluation identifies the importance of a professional graduate breastfeeding and lactation education programme for interdisciplinary health care professionals increasing knowledge, attitudes and practices and ultimately increasing breastfeeding rates in the short and long term, with improved maternal and child health outcomes.

## Introduction

Breastfeeding and lactation are important for maternal, infant and child health. The World Health Organization recommends the initiation of breastfeeding during the first hour following birth, exclusive breastfeeding for the first 6 months of life and continued breastfeeding for two years and beyond, in addition to complementary foods [1]. Breastfeeding maximises protection against short and long term health conditions for both the mother and child [2]. The early breastfeeding phase plays an important part in an infant’s lifelong health and development and is a window of opportunity that healthcare professionals have not embraced fully [3]. A recent Irish national report [4] describes that while 63.1% of infants in Ireland were breastfed at birth only 36.8% of infants were exclusively breastfeeding at discharge from the maternity setting in 2021. At European level, a cross sectional survey from 17 countries reports an exclusive breastfeeding rate of 72.4% at discharge [5]. There are suboptimal breastfeeding rates globally with only 44% of infant’s breastfed exclusively from 0–6 months [1]. Indeed many parents who breastfeed do not reach their personal breastfeeding goals [6].

It is also recognised that breastfeeding rates vary significantly between demographic groups within society. The WHO aims to achieve exclusive breastfeeding rates of 70% by 2030 [7]. Healthcare professionals internationally need to be equipped with the knowledge, practices and attitudes (KAPs) to drive action on infant and young child feeding and support the United Nations Sustainable Development Goals (SDGs). Society has a major impact on breastfeeding practices, therefore, healthcare professionals interacting with society at large are well placed to protect, promote, support and normalise breastfeeding. Along with building the mother-child bond, breastfeeding reduces the risk of many childhood diseases like otitis media, respiratory infections, sudden infant death syndrome (SIDS), diarrhoea, type 1 and 11 diabetes, necrotising enterocolitis, asthma, coeliac disease, crohn’s disease, childhood leukaemia, childhood obesity and it has been linked to a child’s increased intelligence [8–11].

Breastfeeding confers benefits to mothers including weight loss, reducing the risk of type 11 diabetes, osteoporosis, breast and ovarian cancer [8,9,12]. Additionally, breastfeeding and associated social supports reduce the risk of postnatal depression [13]. Breastfeeding therefore supports health and wellbeing and reduces the incidence of illness which has a significant economic advantage to public health services internationally. Investment in breastfeeding and education of interdisciplinary healthcare professionals supports a wider knowledge base within the field of lactation which is influenced by social, political and economic domains [14].

The WHO supports collaboration of multidisciplinary health care professional education as it strengthens health systems and improves patient outcomes. Collaboration within the field of breastfeeding and lactation education enables healthcare professionals to provide comprehensive care for breastfeeding families and may serve as a model to support new lactation curriculums [15]. Interdisciplinary education (IDE) involves a collaboration between two or more professions with participants learning about, from and with each other, primarily in educational and clinical environments [16]. IDE prepares the health care professional to collaborate effectively within multidisciplinary teams [17] it supports achievement of the United Nations Sustainable Development Goals (SDG’s) promoting health and economic growth and reducing inequalities globally. Breastfeeding and lactation education is important for all healthcare professionals to ensure consistent and evidenced based information is provided at each consultation during the perinatal and neonatal period. While barriers to breastfeeding and lactation exist primarily in relation to a lack of support and culture [18,19] a major contribution is the gap in breastfeeding and lactation education provided to healthcare professionals globally that support the breastfeeding dyad on their breastfeeding journey [20,21]. This study describes the achievement of breastfeeding knowledge, attitudes and practices among an interdisciplinary group of health care professionals.

### Professional certificate in breastfeeding and lactation

The Professional Certificate in Breastfeeding and Lactation is a six month graduate programme. It encompasses blended learning, both synchronous and asynchronous learning opportunities, with an indicative student workload of 250 hours. Students attend the university for three full days of learning during the semester, in addition to weekly one hour virtual learning sessions. Independent learning is via the university learning platform, Brightspace. The programme was offered at level 9, National Framework of Qualifications and provides 10 European Credit Transfer System (ECTS). The purpose of the module was to educate healthcare professionals at an advanced level to promote, support and protect breastfeeding for women, infants and families. Participants were nurses, midwives, International Board Certified Lactation Consultants (IBCLC’s), doctors and allied health care professionals.

The aim of this study was to evaluate the KAPs of health care professionals following completion of a university graduate programme for breastfeeding and lactation.

## Methods

### Research design

The study was a descriptive survey design using a validated tool developed by Theodoridis et al. [22] and adapted to assess the KAPs of the multidisciplinary healthcare professional. Permission to use and adapt this validated tool was sought by the authors. The survey was modified and approved by the research team principal investigators (DMcG/NV) who have extensive experience in the topic area and are International Board Certified Lactation Consultants (IBCLC’s).

A pre and post—educational study design was used. All students enrolled on one six month programme were invited to complete an online anonymous survey at two time points January 2023 and July 2023. The study commenced on January 28^th^ 2023 and was completed on August 15^th^ 2023.

Bias was avoided in this study by the anonymised online survey design, by multidisciplinary involvement of the research team and by alignment of UCD Research Ethics Committee Guidelines.

### Survey instrument

The questionnaire comprised four sections. Section One reports demographic characteristics. Section Two included 15 statements to explore knowledge about breastfeeding. Section Three included 16 statements to explore attitudes and perceptions towards breastfeeding. Section Four included 4 questions to explore clinical skills in relation to latch challenges, breast and nipple challenges, supplementation, supporting lactation following preterm birth and suppression of lactation following informed choice or neonatal death. A final question sought information on subsequent education and training requirements and preferred mode of delivery and a comment box was provided for participants to provide additional narrative comments if they wished to expand or elaborate responses. No identifying information was sought and all responses were anonymous.

### Demographics of the sample

Participants were students registered to the Professional Certificate in Breastfeeding and Lactation at a university in Ireland. Students participated in the programme from January to June 2023. The professional participants were all female; nurses, midwives, IBCLC’s, medical doctors and allied healthcare professionals. At the start of the programme in January 2023, fifty- eight students were invited to participate in the pre course survey to ascertain breastfeeding KAPs. A hard copy of the survey was provided in class, which included the participant information form, consent was explained and students engaged with the survey, following informed consent. The data were subsequently transferred from a hard copy to the Qualtrics platform by a research assistant for ease of data analysis.

The response rate of the pre survey was 55 participants representing 36.3%, (n = 20) nurses; 38.1% (n = 21) midwives; and allied healthcare professionals 25.45%, (n = 14). Allied healthcare professionals included doctors, dietitians, physiotherapists and IBCLC’s and results were grouped together to ensure anonymity. The age range of participants was between 30–60 years, with 7.27% (n = 4) under the age of 30 years. Many participants were educated to degree level at 72.73% (n = 40). All participants engaged with the blended learning breastfeeding and lactation programme over a six month period. The indicative content was presented over three separate components: A) Breast physiology and pathology, B) Supporting the breastfeeding dyad and C) Education and communication skills.

Following completion of the programme in June 2023 the post survey was available to all participants via the university email system which included a URL to the Qualtrics survey platform. The post survey established KAPs following the breastfeeding and lactation programme. Participants had access to the survey, study information and written informed consent was provided prior to full participation in the study survey. One reminder was issued to the participants via the university email. The survey was closed on August 15th 2023. The survey was password protected on the computer of the research team.

#### Ethics

Low risk ethical exemption was approved by the Human Research Ethics Committee- Sciences (HREC-LS) in University College Dublin in January 2023 (LS-C-23-17-McGuinness-Vickers).

## Data analysis

The pre survey response was n = 55 (92.82%) and the post survey response rate was n = 33 (60%) with a total combined sample of 88. All students completed the module. This study utilised a pre and post educational intervention model through inferential statistics by use of independent T-tests and comparing mean differences between variables on knowledge, attitude and confidence.

Homogeneity of variance was assessed by Levene’s Test for Equality of Variances. Knowledge and Attitude variables were collapsed from a 5-level categorical variable into binary categorical variables [1.00 (Strongly Agree & Agree); 2.00 (Neither, Disagree & Strongly Disagree)]. Confidence variables are a three-level categorical variable ranging from confident to not confident. All statistical tests were performed and analysed using a 0.05 significance level. Independent T-Tests were carried out as pre, and post survey samples were not equal and respondents had no identifiable variable. Inferential statistics was carried out only on non-IBCLCs. Data were converted into IBM SPSS Ver.29 and a data dictionary was created for ease of statistical analyses.

## Results

Table 1 presents inferential pre and post educational intervention statistics comparing means, mean difference, t-statistic, and associated p-values. Nine statistically significant findings were found in total, relating to those who noted they had no previous training in lactation, all findings showed positive directionality in accordance to learning outcomes of the course.

**Table 1:** Summary of pre and post course assessment on knowledge, attitude and confidence scores between groups.

Summary of pre and post course assessment on Knowledge, Attitude and Confidence scores between groups using independent t-test						
Variable	Group	Pre Course	Post Course	MD (95% CI)	T Stat (df)	p-value
		Mean (SD)	Mean (SD)			
**Knowledge**						
I am confident with my knowledge about breastfeeding	IBCLC	1.00 (0)	1.00 (0)			
	Non-IBCLC	1.33 (0.47)	1.04 (0.20)	0.29 (0.13–0.45)	3.59 (64)	**0.001** ***
I am confident that I can manage breastfeeding-related issues in my everyday practice	IBCLC	1.00 (0)	1.00 (0)			
	Non-IBCLC	1.31 (0.46)	1.04 (0.20)	0.27 (0.11–0.43)	3.37 (64)	**0.001** ***
A carrier of Hepatitis B who has been vaccinated can safely breastfeed	IBCLC	1.00 (<0.00.0)	1.13 (0.35)	-0.12 (-0.42–0.17)	-1.00 (16)	ns
	Non-IBCLC	1.31 (0.46)	1.12 (0.33)	0.19 (-0.001–0.38)	1.98 (63)	**0.05** *
I am confident discussing safe medication use with breastfeeding mothers	IBCLC	1.10 (0.31)	1.13 (0.35)	-0.02 (-0.36–0.31)	-0.15 (16)	ns
	Non-IBCLC	1.69 (0.46)	1.40 (0.50)	0.28 (0.05–0.52)	2.41 (68)	**0.018** **
Breast surgeries i.e., augmentation or reduction make breastfeeding difficult	IBCLC	1.30 (0.48)	1.50 (0.53)	-0.02 (-0.70–0.30)	-0.83 (16)	ns
	Non-IBCLC	1.67 (0.47)	1.32 (0.47)	0.34 (0.10–0.58)	2.91 (68)	**0.005** **
**Attitude**						
Breastfeeding makes the father/partner feel isolated from raising their child	IBCLC	1.90 (0.31)	2.00 (<0.00)	-0.10 (-0.33–0.13)	-0.88 (16)	ns
	Non-IBCLC	1.89 (0.31)	2.00 (<0.00)	-0.11 (-0.20–-0.01)	-2.34 (44)	**0.02** *
**Confidence: How confident you feel helping mothers who are breastfeeding regarding**:						
Latching problems	IBCLC	1.00 (0)	1.00 (0)			
	Non-IBCLC	1.56 (0.50)	1.24 (0.43)	0.31 (0.08–0.54)	2.74 (55.9)	**0.008** **
Recognising and managing nipple problems such as mastitis and nipple thrush	IBCLC	1.10 (0.31)	1.13 (0.35)	-0.02 (-0.36–0.31)	-0.15 (16)	ns
	Non-IBCLC	1.71 (0.43)	1.40 (0.50)	0.31 (0.07–0.54)	2.63 (68)	**0.01** *
Supporting lactation suppression, e.g, following infant loss or maternal decision to stop breastfeeding	IBCLC	1.30 (0.48)	1.13 (0.35)	0.17 (-0.25–0.60)	0.85 (16)	ns
	Non-IBCLC	1.73 (0.4$)	1.44 (0.50)	0.29 (0.04–0.53)	2.41 (44.7)	**0.02** *

SD = Standard Deviation; MD = Mean Difference; CI = Confidence Interval; ns = ot statistically significant; IBCLC = International Board Certified Lactation Consultants.

* Statistically Significance;

** Moderate Statistical Significance;

*** Strong Statistical Significance.

Mean knowledge scores increased following completion of the programme as shown in Table 2; specifically confidence levels in engaging clinically with higher mean knowledge scores associated with the statement “I am confident with my knowledge about breastfeeding” (Mean 1.33 ± 0.47), was significantly higher post course (Mean 1.04 ± 0.20) highlighting expected educational attainment with strong statistically significant mean difference of 0.29 (95% CI, 0.13 to 0.45) (t (64) = 3.59, p = 0.001). Additionally, the knowledge score for “I am confident that I can manage breastfeeding-related issues in my everyday practice” increased (1.31 ± 0.46) post course (1.12 ± 0.33) indicating increased competence in lactation management with statistically significant mean difference of 0.27 (95% CI, 0.11 to 0.43) (t (64) = 3.37, p = 0.001). Increased confidence with breastfeeding was noted by 97.7% (n = 32) of respondents and similarly found for managing issues that arise with breastfeeding for mothers. A sizable majority of respondents 67% (n = 33) reported increased confidence in discussing medication use with people who are breastfeeding. Increased knowledge was confirmed on other key variables following completion of the education programme (Table 1) including awareness of the International Code of Marketing of Breastmilk substitutes.

**Table 2:** Impact of the programme on breastfeeding knowledge.

Knowledge Variables	Group	Pre Course	Post Course	MD (95% CI)	T Stat (df)	p-value
		Mean (SD)	Mean (SD)			
I am confident with my knowledge about breastfeeding	IBCLC	1.00 (0)	1.00 (0)			
	Non-IBCLC	1.33 (0.47)	1.04 (0.20)	0.29 (0.13–0.45)	3.59 (64)	**0.001** ***
Could your level of knowledge be improved	IBCLC	1.00 (<0.00.0)	1.25 (0.46)	-0.25 (-0.63–0.13)	-1.52 (7)	ns
	Non-IBCLC	1.02 (0.14)	1.08 (0.27)	-0.05 (-0.17–0.06)	-0.96 (31.9)	ns
I am confident that I can manage breastfeeding-related issues in my everyday practice	IBCLC	1.00 (0)	1.00 (0)			
	Non-IBCLC	1.31 (0.46)	1.04 (0.20)	0.27 (0.11–0.43)	3.37 (64)	**0.001** ***
Formula milk is easier to digest than maternal milk	IBCLC	2.00 (0)	2.00 (0)			
	Non-IBCLC	2.00 (0)	2.00 (0)			
A breastfeeding mother should avoid alcohol	IBCLC	2.00 (<0.00)	1.88 (0.35)	0.12 (-0.17–0.42)	1.00 (7)	ns
	Non-IBCLC	1.53 (0.50)	1.52 (0.51)	0.01 (-0.23–0.26)	0.10 (68)	ns
A carrier of Hepatitis B who has been vaccinated can safely breastfeed	IBCLC	1.00 (<0.00.0)	1.13 (0.35)	-0.12 (-0.42–0.17)	-1.00 (16)	ns
	Non-IBCLC	1.31 (0.46)	1.12 (0.33)	0.19 (-0.001–0.38)	1.98 (63)	**0.05** *
A carrier of HIV can transfer the virus to her baby through breastfeeding	IBCLC	1.30 (0.48)	1.38 (0.51)	-0.07 (-0.57–0.42)	-0.31 (16)	ns
	Non-IBCLC	1.64 (0.48)	1.44 (0.50)	0.19 (-0.05–0.44)	1.58 (67)	**ns**
A mother with a fever > 38C should temporarily interrupt breastfeeding	IBCLC	2.00 (0)	2.00 (0)			
	Non-IBCLC	1.96 (0.20)	2.00(<0.00)			
A mother with mastitis should stop breastfeeding	IBCLC	2.00 (0)	2.00 (0)			
	Non-IBCLC	1.98 (0.14)	1.96 (0.20)	0.01 (-0.06–0.10)	0.43 (68)	ns
Breastfeeding should continue if the mother smokes	IBCLC	1.20 (0.42)	1.00 (<0.00.0)	0.20 (-0.10–0.50)	1.50 (9)	ns
	Non-IBCLC	1.33 (0.47)	1.16 (0.37)	0.17 (-0.03–0.38)	1.68 (60)	ns
I am confident discussing safe medication use with breastfeeding mothers	IBCLC	1.10 (0.31)	1.13 (0.35)	-0.02 (-0.36–0.31)	-0.15 (16)	ns
	Non-IBCLC	1.69 (0.46)	1.40 (0.50)	0.28 (0.05–0.52)	2.41 (68)	**0.018** **
Breast surgeries i.e., augmentation or reduction make breastfeeding difficult	IBCLC	1.30 (0.48)	1.50 (0.53)	-0.02 (-0.70–0.30)	-0.83 (16)	ns
	Non-IBCLC	1.67 (0.47)	1.32 (0.47)	0.34 (0.10–0.58)	2.91 (68)	**0.005** **
Breastfed babies are less likely to suffer reflux	IBCLC	1.20 (0.42)	1.25 (0.46)	-0.05 (-0.49–0.39)	-0.23 (16)	ns
	Non-IBCLC	1.40 (0.49)	1.24 (0.43)	-0.16 (-0.06–0.38)	1.40 (55)	ns
Breastfeeding mothers need to night wean at 6 months	IBCLC	2.00 (0)	2.00 (0)			
	Non-IBCLC	1.93 (0.25)	2.00(<0.00)	-0.06 (-0.14–0.009)	-1.77 (43)	ns

SD = Standard Deviation; MD = Mean Difference; CI = Confidence Interval; ns = not statistically significant; IBCLC = International Board Certified Lactation Consultants.

* Statistically Significance;

** Moderate Statistical Significance;

*** Strong Statistical Significance.

The impact of the post graduate breastfeeding and lactation programme on attitude and perceptions towards breastfeeding is shown in Table 3. In relation to attitudinal change, statistically significant improvement in considering father or partner was noted with a mean difference of -0.11 (95% CI, -0.20 to -0.01) (t (44) = -2.34, p = 0.02). Almost all respondents reported either breastfeeding their own children or would do in the future. The convenience of breastfeeding was noted by 97% (n = 32). Respondents supported exclusive breastfeeding, 75.6% (n = 25) with 90.9% (n = 30) strongly agreed/agreed that a daily formula milk top up had an impact on exclusive breastfeeding. When asked if a breastfeeding mother should avoid alcohol, the result was mixed with 42% (n = 14) disagreeing/strongly disagreeing and 39% (n = 13) agreed/strongly agreed with the statement.

**Table 3:** Impact of the programme on attitudes and perceptions towards breastfeeding.

Attitude Variables	Group	Pre Course	Post Course	MD (95% CI)	T Stat (df)	p-value
		Mean (SD)	Mean (SD)			
I am in favour of exclusive breastfeeding? (exclusive breastfeeding means no formula milk provided)	IBCLC	1.00 (<0.00)	1.13 (0.35)	-0.42 (-0.48–0.56)	0.16 (15)	ns
	Non-IBCLC	1.04 (0.20)	1.08 (0.27)	-0.03 (-0.15–0.08)	-0.60 (68)	ns
I am in favour of breastfeeding combined with formula milk	IBCLC	1.67 (0.50)	1.63 (0.51)	0.04 (-0.48–0.56)	0.16 (15)	ns
	Non-IBCLC	1.82 (0.39)	1.76 (0.43)	0.05 (-0.14–0.26)	0.57 (67)	ns
I am in favour of breastfeeding in public	IBCLC	1.00 (0)	1.00 (0)			
	Non-IBCLC	1.00 (<0.00)	1.04 (0.20)	-0.04 (-0.12–0.04)	-1.00 (24)	ns
I am in favour of breastfeeding while returning to work	IBCLC	1.00 (0)	1.00 (0)			
	Non-IBCLC	1.02 (0.14)	1.04 (0.20)	-0.01 (-0.10–0.06)	-0.42 (68)	ns
Breastfeeding has an impact on the social life of a mother	IBCLC	1.40 (0.51)	1.50 (0.53)	-0.10 (-0.62–0.42)	-0.40 (16)	ns
	Non-IBCLC	1.42 (0.49)	1.52 (0.51)	-0.09 (-0.34–0.15)	-0.77 (68)	ns
Breastfeeding has an impact on the professional life of a mother	IBCLC	1.40 (0.51)	1.63 (0.51)	-0.22 (-0.74–0.29)	-0.91 (16)	ns
	Non-IBCLC	1.47 (0.50)	1.56 (0.50)	-0.09 (-0.34–0.15)	-0.74 (68)	ns
Breastfeeding makes the father/partner feel isolated from raising their child	IBCLC	1.90 (0.31)	2.00 (<0.00)	-0.10 (-0.33–0.13)	-0.88 (16)	ns
	Non-IBCLC	1.89 (0.31)	2.00 (<0.00)	-0.11 (-0.20–-0.01)	-2.34 (44)	**0.02** *
A daily formula milk top-up has an impact on exclusive breastfeeding	IBCLC	1.20 (0.42)	1.13 (0.35)	0.07 (-0.32–0.47)	0.40 (16)	ns
	Non-IBCLC	1.16 (0.36)	1.08 (0.27)	0.07 (-0.09–0.24)	0.89 (68)	ns
Breastfeeding is more convenient and cheaper than formula milk	IBCLC	1.00 (<0.00)	1.13 (0.35)	-0.12 (-0.42–0.17)	-1.00 (7)	ns
	Non-IBCLC	1.13 (0.34)	1.04 (0.20)	0.09 (-0.03–0.22)	1.43 (67.8)	ns
Mothers with excess milk should be encouraged to donate their milk to maternal/donor milk banks	IBCLC	1.20 (0.42)	1.13 (0.35)	0.07 (-0.32–0.47)	0.40 (16)	ns
	Non-IBCLC	1.29 (0.45)	1.20 (0.40)	0.08 (-0.13–0.30)	0.80 (68)	ns
I have the time to inform antenatal pregnant women about the importance of breastfeeding/risks of not breastfeeding	IBCLC	1.10 (0.31)	1.25 (0.46)	-0.15 (-0.53–0.23)	-0.81 (16)	ns
	Non-IBCLC	1.53 (0.50)	1.44 (0.50)	0.09 (-0.15–0.34)	0.74 (68)	ns
Low breastfeeding rates in Ireland are due to healthcare professionals not informing mothers about breastfeeding	IBCLC	1.70 (0.48)	1.75 (0.46)	-0.05 (-0.52–0.42)	-0.22 (16)	ns
	Non-IBCLC	1.73 (0.44)	1.68 (0.47)	0.05 (-0.17–0.28)	0.46 (68)	ns

SD = Standard Deviation; MD = Mean Difference; CI = confidence Interval; ns = not statistically significant; IBCLC = International Board Certified Lactation Consultants.

* Statistically Significance;

** Moderate Statistical Significance;

*** Strong Statistical Significance.

Following completion of the six month education programme respondents reported higher confidence in skills to support mothers who are breastfeeding regarding latching problems, confidence scores increased from 1.59 ± 0.50 to 1.24 ± 0.43, a significant mean difference of 0.31 (95% CI, 0.08–0.54) with a t(55.9) = 2.74, p = 0.008 (Table 4).

**Table 4:** Impact of the programme on breastfeeding skills.

Confidence: How confident you feel helping mothers who are breastfeeding regarding:	Group	Pre Course	Post Course	MD (95% CI)	T Stat (df)	p-value
		Mean (SD)	Mean (SD)			
Latching problems	IBCLC	1.00 (0)	1.00 (0)			
	Non-IBCLC	1.56 (0.50)	1.24 (0.43)	0.31 (0.08–0.54)	2.74 (55.9)	**0.008** **
Recognising and managing nipple problems such as mastitis and nipple thrush	IBCLC	1.10 (0.31)	1.13 (0.35)	-0.02 (-0.36–0.31)	-0.15 (16)	ns
	Non-IBCLC	1.71 (0.43)	1.40 (0.50)	0.31 (0.07–0.54)	2.63 (68)	**0.01** *
Supporting lactation following preterm birth (Preterm means prior to 37 weeks)	IBCLC	1.10 (0.31)	1.00 (<0.00)	0.10 (-0.13–0.33)	0.88 (16)	ns
	Non-IBCLC	1.69 (0.46)	1.52 (0.51)	0.16 (-0.07–0.40)	1.40 (68)	**ns**
Supporting lactation suppression, e.g, following infant loss or maternal decision to stop breastfeeding	IBCLC	1.30 (0.48)	1.13 (0.35)	0.17 (-0.25–0.60)	0.85 (16)	ns
	Non-IBCLC	1.73 (0.4$)	1.44 (0.50)	0.29 (0.04–0.53)	2.41 (44.7)	**0.02** *

SD = Standard Deviation; MD = Mean Difference; CI = Confidence Interval; ns = Not statistically significant; IBCLC = International Board Certified Lactation Consultants.

* Statistically Significance;

** Moderate Statistical Significance;

*** Strong Statistical Significance.

Respondents noted increased confidence in recognising and managing nipple problems for mothers who are breastfeeding, 82.5% (n = 33) and a sizable majority had improved confidence in managing reflux symptoms 62.5% (n = 25) and over half reported increased confidence to support mothers with lactation suppression following infant loss/ or maternal decision to stop lactation 52.5% (n = 21) (Table 4).

### Continuing professional development and education

In relation to continuing professional development 69.7% (n = 23) were interested in further education. Blended learning was the preferred method of education provision, 87.9% (n = 29); barriers reported included time, 57.6% (n = 19), workload, 45.4% (n = 15) and cost, 42.4% (n = 14).

Some student’s added additional qualitative text to the comment box provided (see Table 5) which supported the quantitative findings in providing a rich insight into their personal experience and further recommendations for the programme.

**Table 5:** Students’ comments on the module.

**Interdisciplinary learning-** *“I really enjoyed the course and the interaction with other professionals”*. *“The blend in healthcare professionals on the course made for interesting discussion and information sharing”*. *“I really enjoyed the course and great to network with different disciplines”*.
**Content interesting and informative- *“****The blended learning was excellent and flexible around working (and family) schedule”*. *“I have taken so much from completing this course that will benefit me in my clinical practice and in educating my colleagues in the future”*. *“It’s been so long since I studied that I was really anxious to start*, *but now I want to keep going”*
**Recommendations—*“****One more face to face day”*. *“If I was to suggest an improvement it might be to create more activity from the start… I would have liked to have got more stuck in from the start*.*”* *“My confidence in breastfeeding sick babies is not as high as other topics and it would be very helpful to have further information on this”*.

## Discussion

Breastfeeding is an immensely fulfilling experience for women and parents, but it can also be physically, socially and psychologically challenging [23,24]. Parents interacting with healthcare professionals during their breastfeeding journey expect consistent information that is evidence based. This study explored the KAPs of an interdisciplinary group of health care professional’s prior to and following a post graduate NFQ Professional Certificate in Breastfeeding and Lactation, within a university setting. Our study identified the importance of a university lactation education programme for interdisciplinary health care professionals, on increasing KAPs and this supports existing literature findings [24]. It is recognised that while midwives at undergraduate level receive education and training to support breastfeeding, there is a lack of formal education in the field of breastfeeding and lactation provided to many healthcare professionals that support the breastfeeding dyad and family, up to two years following birth. This two-year period is important for public health, and the WHO recommends that children are breastfed until 2 years and beyond, with the addition of complementary foods after six months [1]. Importantly public health nurses, midwives and paediatric doctors play a pivotal role in supporting mothers initiate and maintain lactation, providing support with breastfeeding challenges should they arise [25]. Previous research has identified lactation knowledge gaps among health care professionals in the primary and paediatric setting [22,26,27]. Neonatal nurses, paediatric nurses and doctors are faced with the care of more medically challenged infants and children requiring additional lactation support needs.

With low breastfeeding rates internationally there has never been a more optimal time in public health to answer the question posed by Zakarija- Grkovic et al. “Are our babies off to a healthy start?” [28]. Breastfeeding initiation rates are improving, however, there is widespread regional variability. Low breastfeeding rates remain among low income women, teenage mothers and minority women with major implications for maternal and child health outcomes [29]. Given the protective effect of breastfeeding it is a public health issue that requires investment in education and inclusion in related maternal and child health policies and strategies [6].

Increased knowledge and skills among healthcare professionals is imperative to protect the breastfeeding relationship and reduce life course health disparities. Indeed inconsistent information provided by healthcare providers has long been identified as a barrier to successful breastfeeding [30]. A recent study by Durocher and Ralph [31] offers the opinion that inadequate knowledge of healthcare professionals, over reliance on the International Board Certified Lactation Consultant (IBCLC) and complacency with early breastfeeding cessation are factors responsible for low breastfeeding rates in the community and paediatric setting.

The mode of programme delivery was fundamental to the success of the graduate breastfeeding education programme, with participants reporting the accessibility of a blended learning approach, particularly HCP’s employed full time, amid staff shortages within the health services generally. E-learning was an important part of the programme and this learning method has been identified as comparable to face-to face teaching especially in terms of knowledge and student satisfaction [32]. Sandhi et al [24] suggest clinical practicums and skills lab teaching instil deep knowledge and skills and indeed this was evident both during the programme and in the study findings. Respondents in our study reported increased confidence in managing breastfeeding challenges such as latch issues, nipple pain, and management of reflux in the breastfed infant and medication and breastfeeding. Interprofessional education and learning was positive during the programme and enabled better collaboration between healthcare professionals [33]. The integrated position of the IBCLC with the multidisciplinary health care professional class was innovative. It recognised that the IBCLC supports the wider interdisciplinary team, enriching the breastfeeding experience of mothers and families [34]. The results of this study may be generalisable to other populations and offers potential for further research.

Notwithstanding the benefits of the programme the duration of six months was challenging to meet the module learning outcomes and prepare students for all inevitabilities that can arise within the field of breastfeeding and lactation. Comparison of the pre and post questionnaire report nine statistically significant results following completion of the university breastfeeding and lactation programme with knowledge scores increasing specifically in relation to managing breastfeeding related issues in everyday practice for the health care professionals, in addition to breastfeeding and surgery, and awareness of the International Code of Marketing of Breastmilk Substitutes. It is imperative that healthcare professionals supporting the breastfeeding dyad have a greater understanding of normal newborn behaviours as an expected phases of human growth and development, providing guidance and assisting in finding breastfeeding solutions for breastfeeding challenges [35]. The recent Lancet series on breastfeeding highlights the importance of education for healthcare professionals free from the commercial influences, to facilitate exclusive breastfeeding [35].

Participants that engaged with the programme were motivated and took responsibility for their learning, investing time and energy to ensure that their increased knowledge supported future breastfeeding parents receive timely, accurate and individualised support. The ultimate goal of any further breastfeeding educational programme is to drive action on infant and young child feeding and support the United Nations SDG’s [36] and ensure the best start in life for every child.

## Limitations

There are some limitations for this study as it reports data from one cohort of students and the sample sizes are small. The data are not linked and it is recognised that allocating a random study ID, could be used in subsequent evaluations. There is potential for further research including a post-test questionnaire following a longer period to establish continuing knowledge retention and breastfeeding rates. This evaluation provides useful data in the ongoing professional development and education for healthcare professionals and for this critical public health issue to support mothers and families and improve health outcomes at population level. There is scope to increase the programme content in relation to breastfeeding and the sick neonate.

## Conclusion

Historically breastfeeding education programmes are provided to both midwives and public health nurses. There is a gap in breastfeeding and lactation education provided to the multidisciplinary healthcare professionals directly supporting the breastfeeding dyad and this has implications for public health. This lack of education has been identified as a major factor in relation to low breastfeeding rates.

Our research identified nine statistically significant results including increased knowledge, improved attitude and confidence in skills to support breastfeeding mothers, following completion of the post graduate breastfeeding and lactation programme. Increased confidence was reported in relation to latch challenges, managing nipple problems and reflux and breastfeeding. The findings have implications for improved maternal and child health policy, reaffirming the importance of breastfeeding and lactation programmes for the multidisciplinary team who support women and children during the perinatal and neonatal period.

## Supporting information

S1 Table Summary of pre and post course assessment on knowledge, attitude and confidence scores between groups. (DOCX)

S2 Table Impact of the programme on breastfeeding knowledge. (DOCX)

S3 Table Impact of the programme on attitude and perception towards breastfeeding. (DOCX)

S4 Table Impact of the programme on breastfeeding skills. (DOCX)

S5 Table Student’s comments on the module. (DOCX)
