# Supplementary material for: Evaluating interdisciplinary breastfeeding and lactation knowledge, attitudes and skills: An evaluation of a professional graduate programme for healthcare professionals
Source: PLoS One. 2025 Jan 31;20(1):e0310500. doi: 10.1371/journal.pone.0310500 (PMC11785295; doi:10.1371/journal.pone.0310500)
Supplement: S1 Table — (DOCX) [file pone.0310500.s001.docx]

**S1 Table : Summary of Pre and Post Course Assessment on Knowledge, Attitude and Confidence Scores between groups**

| **Summary of pre and post course assessment on Knowledge, Attitude and Confidence scores between groups using independent t-test** | | | | | | | | | | | | | |
| --- | --- | --- | --- | --- | --- | --- | --- | --- | --- | --- | --- | --- | --- |
| **Variable** | **Group** | **Pre Course** | | | | **Post Course** | **MD (95% CI)** | | **T Stat (df)** | | | **p-value** | |
|  |  | **Mean (SD)** | | | | **Mean (SD)** |  |  |  |  |  |  |  |
| **Knowledge** | | | | | | | | | | | | | |
| I am confident with my knowledge about breastfeeding | IBCLC | 1.00 (0) | | | | 1.00 (0) |  | |  | | |  | |
|  | Non-IBCLC | 1.33 (0.47) | | | | 1.04 (0.20) | 0.29 (0.13 - 0.45) | | 3.59 (64) | | | **0.001***** | |
| I am confident that I can manage breastfeeding-related issues in my everyday practice | IBCLC | 1.00 (0) | | | | 1.00 (0) |  | |  | | |  | |
|  | Non-IBCLC | 1.31 (0.46) | | | | 1.04 (0.20) | 0.27 (0.11 - 0.43) | | 3.37 (64) | | | **0.001***** | |
| A carrier of Hepatitis B who has been vaccinated can safely breastfeed | IBCLC | 1.00 (<0.00.0) | | | | 1.13 (0.35) | -0.12 (-0.42 - 0.17) | | -1.00 (16) | | | ns | |
|  | Non-IBCLC | 1.31 (0.46) | | | | 1.12 (0.33) | 0.19 (-0.001 - 0.38) | | 1.98 (63) | | | **0.05*** | |
| I am confident discussing safe medication use with breastfeeding mothers | IBCLC | 1.10 (0.31) | | | | 1.13 (0.35) | -0.02 (-0.36-0.31) | | -0.15 (16) | | | ns | |
|  | Non-IBCLC | 1.69 (0.46) | | | | 1.40 (0.50) | 0.28 (0.05 - 0.52) | | 2.41 (68) | | | **0.018**** | |
| Breast surgeries i.e., augmentation or reduction make breastfeeding difficult | IBCLC | 1.30 (0.48) | | | | 1.50 (0.53) | -0.02 (-0.70-0.30) | | -0.83 (16) | | | ns | |
|  | Non-IBCLC | 1.67 (0.47) | | | | 1.32 (0.47) | 0.34 (0.10 - 0.58) | | 2.91 (68) | | | **0.005**** | |
| **Attitude** | | | | | | | | | | | | | |
| Breastfeeding makes the father/partner feel isolated from raising their child | IBCLC | 1.90 (0.31) | | | | 2.00 (<0.00) | -0.10 (-0.33 - 0.13) | | -0.88 (16) | | | ns | |
|  | Non-IBCLC | 1.89 (0.31) | | | | 2.00 (<0.00) | -0.11 (-0.20 - -0.01) | | -2.34 (44) | | | **0.02*** | |
| **Confidence: How confident you feel helping mothers who are breastfeeding regarding:** | | | | | | | | | | | | | |
| Latching problems | IBCLC | | 1.00 (0) | 1.00 (0) | | |  | | | |  | |  |
|  | Non-IBCLC | | 1.56 (0.50) | 1.24 (0.43) | | | 0.31 (0.08 - 0.54) | 2.74 (55.9) | | | | **0.008**** | |
| Recognising and managing nipple problems such as mastitis and nipple thrush | IBCLC | | 1.10 (0.31) | 1.13 (0.35) | | | -0.02 (-0.36 - 0.31) | -0.15 (16) | | | | ns | |
|  | Non-IBCLC | | 1.71 (0.43) | 1.40 (0.50) | | | 0.31 (0.07 - 0.54) | 2.63 (68) | | | | **0.01*** | |
| Supporting lactation suppression, e.g, following infant loss or maternal decision to stop breastfeeding | IBCLC | | 1.30 (0.48) | 1.13 (0.35) | | | 0.17 (-0.25 - 0.60) | 0.85 (16) | | | | ns | |
|  | Non-IBCLC | | 1.73 (0.4$) | 1.44 (0.50) | | | 0.29 (0.04 - 0.53) | 2.41 (44.7) | | | | **0.02*** | |
| **SD = Standard Deviation; MD = Mean Difference; CI = Confidence Interval; ns = ot statistically significant; IBCLC = International Board Certified Lactation Consultants** | | | | | | | | | | | | | |
| *** Statistically Significance; ** Moderate Statistical Significance; *** Strong Statistical Significance** | | | | |  | |  | | |  | |  | |
