# Supplementary material for: Evaluating interdisciplinary breastfeeding and lactation knowledge, attitudes and skills: An evaluation of a professional graduate programme for healthcare professionals
Source: PLoS One. 2025 Jan 31;20(1):e0310500. doi: 10.1371/journal.pone.0310500 (PMC11785295; doi:10.1371/journal.pone.0310500)
Supplement: S2 Table — (DOCX) [file pone.0310500.s002.docx]

**S2 Table : Impact of the programme on breastfeeding knowledge**

| **Knowledge Variables** | **Group** | **Pre Course** | **Post Course** | **MD (95% CI)** | **T Stat (df)** | **p-value** |
| --- | --- | --- | --- | --- | --- | --- |
|  |  | **Mean (SD)** | **Mean (SD)** |  |  |  |
| I am confident with my knowledge about breastfeeding | IBCLC | 1.00 (0) | 1.00 (0) |  |  |  |
|  | Non-IBCLC | 1.33 (0.47) | 1.04 (0.20) | 0.29 (0.13 - 0.45) | 3.59 (64) | **0.001***** |
| Could your level of knowledge be improved | IBCLC | 1.00 (<0.00.0) | 1.25 (0.46) | -0.25 (-0.63 - 0.13) | -1.52 (7) | ns |
|  | Non-IBCLC | 1.02 (0.14) | 1.08 (0.27) | -0.05 (-0.17 - 0.06) | -0.96 (31.9) | ns |
| I am confident that I can manage breastfeeding-related issues in my everyday practice | IBCLC | 1.00 (0) | 1.00 (0) |  |  |  |
|  | Non-IBCLC | 1.31 (0.46) | 1.04 (0.20) | 0.27 (0.11 - 0.43) | 3.37 (64) | **0.001***** |
| Formula milk is easier to digest than maternal milk | IBCLC | 2.00 (0) | 2.00 (0) |  |  |  |
|  | Non-IBCLC | 2.00 (0) | 2.00 (0) |  |  |  |
| A breastfeeding mother should avoid alcohol | IBCLC | 2.00 (<0.00) | 1.88 (0.35) | 0.12 (-0.17 - 0.42) | 1.00 (7) | ns |
|  | Non-IBCLC | 1.53 (0.50) | 1.52 (0.51) | 0.01 (-0.23 - 0.26) | 0.10 (68) | ns |
| A carrier of Hepatitis B who has been vaccinated can safely breastfeed | IBCLC | 1.00 (<0.00.0) | 1.13 (0.35) | -0.12 (-0.42 - 0.17) | -1.00 (16) | ns |
|  | Non-IBCLC | 1.31 (0.46) | 1.12 (0.33) | 0.19 (-0.001 - 0.38) | 1.98 (63) | **0.05*** |
| A carrier of HIV can transfer the virus to her baby through breastfeeding | IBCLC | 1.30 (0.48) | 1.38 (0.51) | -0.07 (-0.57 - 0.42) | -0.31 (16) | ns |
|  | Non-IBCLC | 1.64 (0.48) | 1.44 (0.50) | 0.19 (-0.05 - 0.44) | 1.58 (67) | **ns** |
| A mother with a fever > 38C should temporarily interrupt breastfeeding | IBCLC | 2.00 (0) | 2.00 (0) |  |  |  |
|  | Non-IBCLC | 1.96 (0.20) | 2.00(<0.00) |  |  |  |
| A mother with mastitis should stop breastfeeding | IBCLC | 2.00 (0) | 2.00 (0) |  |  |  |
|  | Non-IBCLC | 1.98 (0.14) | 1.96 (0.20) | 0.01 (-0.06 - 0.10) | 0.43 (68) | ns |
| Breastfeeding should continue if the mother smokes | IBCLC | 1.20 (0.42) | 1.00 (<0.00.0) | 0.20 (-0.10 - 0.50) | 1.50 (9) | ns |
|  | Non-IBCLC | 1.33 (0.47) | 1.16 (0.37) | 0.17 (-0.03 - 0.38) | 1.68 (60) | ns |
| I am confident discussing safe medication use with breastfeeding mothers | IBCLC | 1.10 (0.31) | 1.13 (0.35) | -0.02 (-0.36-0.31) | -0.15 (16) | ns |
|  | Non-IBCLC | 1.69 (0.46) | 1.40 (0.50) | 0.28 (0.05 - 0.52) | 2.41 (68) | **0.018**** |
| Breast surgeries i.e., augmentation or reduction make breastfeeding difficult | IBCLC | 1.30 (0.48) | 1.50 (0.53) | -0.02 (-0.70-0.30) | -0.83 (16) | ns |
|  | Non-IBCLC | 1.67 (0.47) | 1.32 (0.47) | 0.34 (0.10 - 0.58) | 2.91 (68) | **0.005**** |
| Breastfed babies are less likely to suffer reflux | IBCLC | 1.20 (0.42) | 1.25 (0.46) | -0.05 (-0.49 - 0.39) | -0.23 (16) | ns |
|  | Non-IBCLC | 1.40 (0.49) | 1.24 (0.43) | -0.16 (-0.06 - 0.38) | 1.40 (55) | ns |
| Breastfeeding mothers need to night wean at 6 months | IBCLC | 2.00 (0) | 2.00 (0) |  |  |  |
|  | Non-IBCLC | 1.93 (0.25) | 2.00(<0.00) | -0.06 (-0.14 - 0.009) | -1.77 (43) | ns |
| **SD = Standard Deviation; MD = Mean Difference; CI = Confidence Interval; ns = not statistically significant; IBCLC = International Board Certified Lactation Consultants** | | | | | |  |
| *** Statistically Significance; ** Moderate Statistical Significance; *** Strong Statistical Significance** | |  |  |  |  |  |
