# Supplementary material for: Evaluating interdisciplinary breastfeeding and lactation knowledge, attitudes and skills: An evaluation of a professional graduate programme for healthcare professionals
Source: PLoS One. 2025 Jan 31;20(1):e0310500. doi: 10.1371/journal.pone.0310500 (PMC11785295; doi:10.1371/journal.pone.0310500)
Supplement: S3 Table — (DOCX) [file pone.0310500.s003.docx]

**S 3 Table: Impact of the programme on attitude and perception towards breastfeeding**

| **Attitude Variables** | **Group** | **Pre Course** | **Post Course** | **MD (95% CI)** | **T Stat (df)** | | **p-value** | |
| --- | --- | --- | --- | --- | --- | --- | --- | --- |
|  |  | **Mean (SD)** | **Mean (SD)** |  |  |  |  |  |
| I am in favour of exclusive breastfeeding? (exclusive breastfeeding means no formula milk provided) | IBCLC | 1.00 (<0.00) | 1.13 (0.35) | -0.42 (-0.48 - 0.56) | 0.16 (15) | | ns | |
|  | Non-IBCLC | 1.04 (0.20) | 1.08 (0.27) | -0.03 (-0.15 - 0.08) | -0.60 (68) | | ns | |
| I am in favour of breastfeeding combined with formula milk | IBCLC | 1.67 (0.50) | 1.63 (0.51) | 0.04 (-0.48 - 0.56) | 0.16 (15) | | ns | |
|  | Non-IBCLC | 1.82 (0.39) | 1.76 (0.43) | 0.05 (-0.14 - 0.26) | 0.57 (67) | | ns | |
| I am in favour of breastfeeding in public | IBCLC | 1.00 (0) | 1.00 (0) |  |  | |  | |
|  | Non-IBCLC | 1.00 (<0.00) | 1.04 (0.20) | -0.04 (-0.12 - 0.04) | -1.00 (24) | | ns | |
| I am in favour of breastfeeding while returning to work | IBCLC | 1.00 (0) | 1.00 (0) |  |  | |  | |
|  | Non-IBCLC | 1.02 (0.14) | 1.04 (0.20) | -0.01 (-0.10 - 0.06) | -0.42 (68) | | ns | |
| Breastfeeding has an impact on the social life of a mother | IBCLC | 1.40 (0.51) | 1.50 (0.53) | -0.10 (-0.62 - 0.42) | -0.40 (16) | | ns | |
|  | Non-IBCLC | 1.42 (0.49) | 1.52 (0.51) | -0.09 (-0.34 - 0.15) | -0.77 (68) | | ns | |
| Breastfeeding has an impact on the professional life of a mother | IBCLC | 1.40 (0.51) | 1.63 (0.51) | -0.22 (-0.74 - 0.29) | -0.91 (16) | | ns | |
|  | Non-IBCLC | 1.47 (0.50) | 1.56 (0.50) | -0.09 (-0.34 - 0.15) | -0.74 (68) | | ns | |
| Breastfeeding makes the father/partner feel isolated from raising their child | IBCLC | 1.90 (0.31) | 2.00 (<0.00) | -0.10 (-0.33 - 0.13) | -0.88 (16) | | ns | |
|  | Non-IBCLC | 1.89 (0.31) | 2.00 (<0.00) | -0.11 (-0.20 - -0.01) | -2.34 (44) | | **0.02*** | |
| A daily formula milk top-up has an impact on exclusive breastfeeding | IBCLC | 1.20 (0.42) | 1.13 (0.35) | 0.07 (-0.32 - 0.47) | 0.40 (16) | | ns | |
|  | Non-IBCLC | 1.16 (0.36) | 1.08 (0.27) | 0.07 (-0.09 - 0.24) | 0.89 (68) | | ns | |
| Breastfeeding is more convenient and cheaper than formula milk | IBCLC | 1.00 (<0.00) | 1.13 (0.35) | -0.12 (-0.42 - 0.17) | -1.00 (7) | | ns | |
|  | Non-IBCLC | 1.13 (0.34) | 1.04 (0.20) | 0.09 (-0.03 - 0.22) | 1.43 (67.8) | | ns | |
| Mothers with excess milk should be encouraged to donate their milk to maternal/donor milk banks | IBCLC | 1.20 (0.42) | 1.13 (0.35) | 0.07 (-0.32 - 0.47) | 0.40 (16) | | ns | |
|  | Non-IBCLC | 1.29 (0.45) | 1.20 (0.40) | 0.08 (-0.13 - 0.30) | 0.80 (68) | | ns | |
| I have the time to inform antenatal pregnant women about the importance of breastfeeding/risks of not breastfeeding | IBCLC | 1.10 (0.31) | 1.25 (0.46) | -0.15 (-0.53 - 0.23) | -0.81 (16) | | ns | |
|  | Non-IBCLC | 1.53 (0.50) | 1.44 (0.50) | 0.09 (-0.15 - 0.34) | 0.74 (68) | | ns | |
| Low breastfeeding rates in Ireland are due to healthcare professionals not informing mothers about breastfeeding | IBCLC | 1.70 (0.48) | 1.75 (0.46) | -0.05 (-0.52 - 0.42) | -0.22 (16) | | ns | |
|  | Non-IBCLC | 1.73 (0.44) | 1.68 (0.47) | 0.05 (-0.17 - 0.28) | 0.46 (68) | | ns | |
| **SD = Standard Deviation; MD = Mean Difference; CI = confidence Interval; ns = not statistically significant; IBCLC = International Board Certified Lactation Consultants** | | | | | | | |  |
| *** Statistically Significance; ** Moderate Statistical Significance; *** Strong Statistical Significance** | | | | | |  |  | |
