# Supplementary material for: Evaluating interdisciplinary breastfeeding and lactation knowledge, attitudes and skills: An evaluation of a professional graduate programme for healthcare professionals
Source: PLoS One. 2025 Jan 31;20(1):e0310500. doi: 10.1371/journal.pone.0310500 (PMC11785295; doi:10.1371/journal.pone.0310500)
Supplement: S4 Table — (DOCX) [file pone.0310500.s004.docx]

**S 4 Table: Impact of the programme on breastfeeding skills**

| **Confidence: How confident you feel helping mothers who are breastfeeding regarding:** | **Group** | **Pre Course** | **Post Course** | **MD (95% CI)** | **T Stat (df)** | **p-value** | |
| --- | --- | --- | --- | --- | --- | --- | --- |
|  |  | **Mean (SD)** | **Mean (SD)** |  |  |  |  |
| Latching problems | IBCLC | 1.00 (0) | 1.00 (0) |  |  |  | |
|  | Non-IBCLC | 1.56 (0.50) | 1.24 (0.43) | 0.31 (0.08 - 0.54) | 2.74 (55.9) | **0.008**** | |
| Recognising and managing nipple problems such as mastitis and nipple thrush | IBCLC | 1.10 (0.31) | 1.13 (0.35) | -0.02 (-0.36 - 0.31) | -0.15 (16) | ns | |
|  | Non-IBCLC | 1.71 (0.43) | 1.40 (0.50) | 0.31 (0.07 - 0.54) | 2.63 (68) | **0.01*** | |
| Supporting lactation following preterm birth (Preterm means prior to 37 weeks) | IBCLC | 1.10 (0.31) | 1.00 (<0.00) | 0.10 (-0.13 - 0.33) | 0.88 (16) | ns | |
|  | Non-IBCLC | 1.69 (0.46) | 1.52 (0.51) | 0.16 (-0.07 - 0.40) | 1.40 (68) | **ns** | |
| Supporting lactation suppression, e.g, following infant loss or maternal decision to stop breastfeeding | IBCLC | 1.30 (0.48) | 1.13 (0.35) | 0.17 (-0.25 - 0.60) | 0.85 (16) | ns | |
|  | Non-IBCLC | 1.73 (0.4$) | 1.44 (0.50) | 0.29 (0.04 - 0.53) | 2.41 (44.7) | **0.02*** | |
| **SD = Standard Deviation; MD = Mean Difference; CI = Confidence Interval; ns = Not statistically significant; IBCLC = International Board Certified Lactation Consultants** | | | | | | |  |
| *** Statistically Significance; ** Moderate Statistical Significance; *** Strong Statistical Significance** | | | | | | |  |
