# Supplementary material for: Evaluating interdisciplinary breastfeeding and lactation knowledge, attitudes and skills: An evaluation of a professional graduate programme for healthcare professionals
Source: PLoS One. 2025 Jan 31;20(1):e0310500. doi: 10.1371/journal.pone.0310500 (PMC11785295; doi:10.1371/journal.pone.0310500)
Supplement: S5 Table — (DOCX) [file pone.0310500.s005.docx]

**S 5 Table Student’s comments on the module**

| **Interdisciplinary learning*-*** *“I really enjoyed the course and the interaction with other professionals”.*  *“The blend in healthcare professionals on the course made for interesting discussion and information sharing”.*  *“I really enjoyed the course and great to network with different disciplines”.* |
| --- |
| **Content interesting and informative- *“****The blended learning was excellent and flexible around working (and family) schedule”.*  *“I have taken so much from completing this course that will benefit me in my clinical practice and in educating my colleagues in the future”.*  *“It’s been so long since I studied that I was really anxious to start, but now I want to keep going”* |
| **Recommendations *- “****One more face to face day”.*  *“If I was to suggest an improvement it might be to create more activity from the start… I would have liked to have got more stuck in from the start.”*  *“My confidence in breastfeeding sick babies is not as high as other topics and it would be very helpful to have further information on this”.* |
